# A disintegrin and metalloproteinase 17 mRNA and protein expression in esophageal squamous cell carcinoma, as well as its clinicopathological factors and prognosis

**DOI:** 10.3892/mmr.2014.2802

**Published:** 2014-10-29

**Authors:** HONG-BIN LIU, QI-CHANG YANG, YI SHEN, YAN ZHU, XIAO-JUAN ZHANG, HAO CHEN

**Affiliations:** Department of Pathology, The Second Affiliated Hospital of Nantong University, Nantong, Jiangsu 226001, P.R. China

**Keywords:** esophageal cancer, a disintegrin and metalloproteinase 17 mRNA, reverse transcription polymerase chain reaction, immunohistochemistry, prognosis

## Abstract

The aim of the present study was to explore a disintegrin and metalloproteinase 17 (ADAM17) mRNA and protein expression in esophageal squamous cell carcinoma and its association with clinicopathological factors and prognosis. Through semi-quantitative reverse transcription polymerase chain reaction, the ADAM17 mRNA expression in 50 cases of esophageal squamous cell carcinoma and corresponding normal esophageal mucosa were detected. Using streptavidin peroxidase conjugated immunohistochemistry, ADAM17 protein levels were detected in 80 cases of esophageal squamous cell carcinoma and corresponding normal esophageal mucosa. A log rank test and the Cox proportional hazards model were used for the esophageal cancer survival analysis. ADAM17 mRNA expression levels in esophageal squamous cell carcinoma and corresponding normal esophageal mucosa were 0.937±0.241 and 0.225±0.077, respectively (P<0.01). ADAM17 mRNA expression in esophageal squamous cell carcinoma was correlated with lymph node metastasis (P<0.01) and tumor, node and metastasis (TNM) staging (P<0.05), however, it was not correlated with gender, age or histological grade (P>0.05). ADAM17 protein expression rates in esophageal squamous cell carcinoma and corresponding normal esophageal mucosa were 66.25 and 6.25% respectively, a difference that was statistically significant (P<0.01). In addition, ADAM17 protein expression in esophageal squamous cells was correlated with lymph node metastasis and TNM stage (P<0.05), while it was not correlated with gender, age or histological grade (P>0.05). ADAM17 protein expression and epidermal growth factor receptor (EGFR) protein expression were positively correlated (P<0.01). Lymph node metastasis, TNM stage, ADAM17 and EGFR protein expression may be used as independent prognostic indicators of esophageal squamous cell carcinoma (all P<0.05). ADAM17 mRNA and protein were highly expressed in esophageal squamous cell carcinoma; they have important roles in invasion and metastasis and a certain value in judging the prognosis of patients with esophageal squamous cell carcinoma.

## Introduction

Esophageal cancer is one of the top ten most common types of cancer in human beings, with operable esophageal cancer patients accounting for only 20% of patients. The survival rate is <10% after five years; the primary reason for the poor prognosis of esophageal cancer patients is the aggressiveness of tumor towards surrounding healthy tissue and the ease at which it metastasizes to the lymph nodes. Although there have been numerous studies on the molecular markers of esophageal cancer, the underlying mechanisms of invasion and metastasis remain to be determined. As an important member of the a disintegrin and metalloproteinase (ADAM) family, ADAM17, termed tumor necrosis factor-α converting enzyme (TACE), is the primary secretase responsible for releasing the soluble form of tumor necrosis factor-α from the plasma membrane and is the primary sheddase for multiple epidermal growth factor receptor (EGFR) pro-ligands (1,2). The function of the ADAMl7 protein is the hydrolysis and release of precursor cell surface proteins. It transversely activates cell surface molecules of the cell signaling pathway in order to alter the signal transmission in the tumor microenvironment, which associated with tumorigenesis and tumor progression (3–5). ADAM17 is involved in proteolysis of collagen V, VII and X, and gelatin, as well as the release of certain integrins from the cell surface. This suggests that ADAM17 may affect tumor angiogenesis and the invasive activity of tumor cells (1). Recent studies have shown an increase in the expression and activity of ADAM17 in a number of human tumor tissues, including breast cancer, lung cancer, gastric cancer and liver cancer (6–9). However, there are limited studies reporting the expression of ADAM17 in esophageal cancer. An improved understanding of the importance of ADAM17 and the downstream EGFR signaling pathway in esophageal cancer invasion may aid in the development of therapeutic strategies aimed at reducing the invasiveness of esophageal cancer.

The current study used the reverse transcription polymerase chain reaction (RT-PCR) technique and streptavidin peroxidase conjugated (SP) immunohistochemistry to detect ADAMl7 mRNA and protein expression in esophageal squamous cell carcinoma in order to explore its clinical pathological significance and prognostic value in patients with esophageal squamous cell carcinoma.

## Materials and methods

### Clinical pathological material

Between 2007 and 2010 in the Pathology Department of the First People’s Hospital of Nantong, esophageal squamous cell carcinoma and normal esophageal mucosa specimens were obtained from 50 patients and prepared for RT-PCR detection. The specimens consisted of fresh tissues without necrosis; 30 min following the extraction of the specimens they were placed into liquid nitrogen and quickly transferred to a refrigerator where they were maintained at −80°C prior to use. None of the patients received pre-operative radiotherapy or chemotherapy, and the cases were diagnosed by histopathological examination. The patients included 33 males and 17 females with ages ranging from 45 to 77 years, and a median age of 61 years. The patients were spread among all phases of tumor, node and metastasis (TNM) classification: phase I (n=5), phase II (n=24), phase III (n=15) and phase IV (n=6).

From 2000 to 2002, esophageal squamous cell carcinoma and normal esophageal mucosa specimens were obtained from 80 patients and subjected to SP immunohistochemistry. Patients included 57 males and 23 females with ages ranging from 47 to 75 years, and a median age of 61 years. The patients were spread among all phases of TNM classification: phase I (n=5), phase II (n=38), phase III (n=31) and phase IV (n=6). No patients had a history of preoperative radiotherapy or chemotherapy. Of the 80 esophageal squamous cell carcinoma patients who received a follow-up letter, 16 cases were missing and 64 cases replied to the follow-up, giving a follow-up rate of 80% (64/80). The follow-up time was >60 months. The study was approved by the ethics committee of the Second Affiliated Hospital of Nantong University (Nantong, China). Written informed consent was obtained from all participants.

### ADAMl7 mRNA detection using RT-PCR

Total RNA extraction was carried out as follows: 50 mg tissue homogenates were collected, and the total tissue RNA was extracted using TRIzol^®^ (Invitrogen Life Technologies, Carlsbad, CA, USA). The purity of a sample of the total RNA was measured using an ultraviolet spectrophotometer (Beijing Purkinje General Instrument Co., Ltd., Beijing, China).

ADAM17 mRNA primers were designed according to the literature (1), and the size of their product was 440 bp. The primer sequences were as follows: forward, 5′-GCACAGGTAATAGCAGTGAGTGC-3′, and reverse, 5′-CACACAATGGACAAGAATGCTG-3′ for ADAM17 mRNA; and forward, 5′-TTCCAGCCTTCCTTCCTGG-3′, and reverse, 5′-TTGCGCTCAGGAGGAGCAAT-3′ for β-actin. ADAM17 mRNA and β-actin primers were synthesized by Shanghai Biotechnology Company (Shanghai, China). The ADAM17 and β-actin reaction were performed within a tube, and the conditions for gene amplification were as follows: 95°C denaturation for 5 min, followed by 40 cycles of 94°C for 50 sec, 52°C for 1 min and 72°C for 1 min.

### Semi-quantification of PCR results

PCR products were transferred onto a 1.5% agarose gel for electrophoresis, the Eagle Eye™ II Image Analysis system (Agilent Technologies Inc., Santa Clara, CA, USA) was used to scan the bands of the amplified products. The ratio of the optical density of ADAM17 to the optical density of β-actin in the same tube was used to represent the ADAM17 mRNA expression.

### ADAM17 and EGFR protein detection with SP immunohistochemistry

All specimens were fixed in 10% neutral formalin, paraffin-embedded and sliced (thickness, 4 μm). Hematoxylin and eosin (HE)staining and immunohistochemical SP staining were then performed. The primary anti-ADAM17 and EGFR antibodies were mouse anti-human monoclonal antibodies purchased from Beijing Zhongshan Golden Bridge Company (Beijing, China). According to the literature (2), based on the percentage of positive cells, the cells were divided into four groups, 0: no positive cells, 1 point: <30% positive cells, 2 points: 30–70% positive cells, 3 points: >70% positive cells. The cells were subsequently divided into further groups according to the staining intensity, 0 point: no staining, 1 point: positive cells were stained pale yellow; 2 points: positive cells were stained yellow and 3 points: positive cells were stained brown. The two combined scores were taken as the final result: 0 point (−), 1 to 2 points (+), 3 to 4 points (+ +), 5 to 6 points (+ + +). In statistics, the (−) and (+) were classified as the negative group, (+ +) and (+ + +) were classified as the positive group.

### Statistical analysis

The Stata7.0 statistical package was used for all statistical analysis. ADAM17 mRNA expression levels were expressed as the mean ± standard deviation (χ̄ ± s). The Student’s t test was used for comparison in different groups. The rates were compared with a χ^2^ test. The univariate analysis of the follow-up results was performed with a logrank test, and Kaplan-Meier survival curves were created. Multivariate survival analysis was performed using Cox proportional hazard model statistics. P<0.05 was considered to indicate a statistically significant difference.

## Results

### ADAM17 mRNA expression of esophageal squamous cell carcinoma

**Figure 1:**
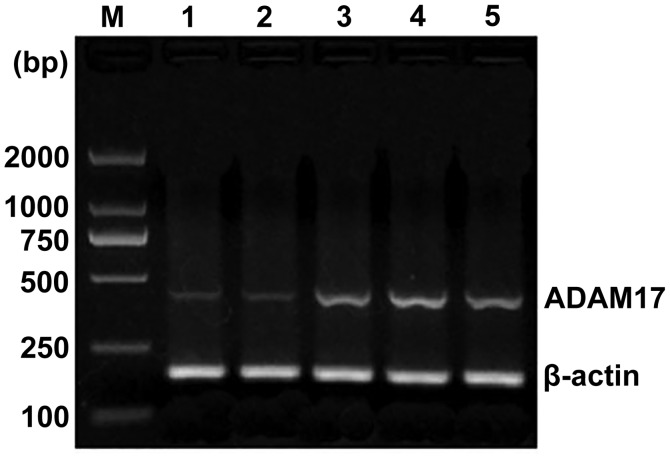
A disintegrin and metalloproteinase 17 mRNA reverse transcription polymerase chain reaction amplification results. Lanes: M, DNA marker; 1 and 2, normal esophageal mucosa; 3–5, esophageal squamous cell carcinoma.

**Figure 2:**
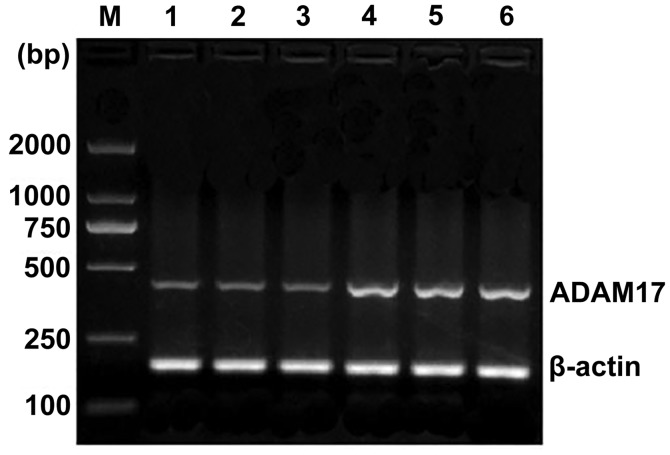
ADAM17 mRNA reverse transcription polymerase chain reaction amplification results. Lanes: M, DNA marker; 1–3, esophageal squamous cell carcinoma without lymph node metastasis; 4–6, esophageal squamous cell carcinoma with lymph node metastasis. ADAM17, a disintegrin and metalloproteinase 17.

**Figure 3:**
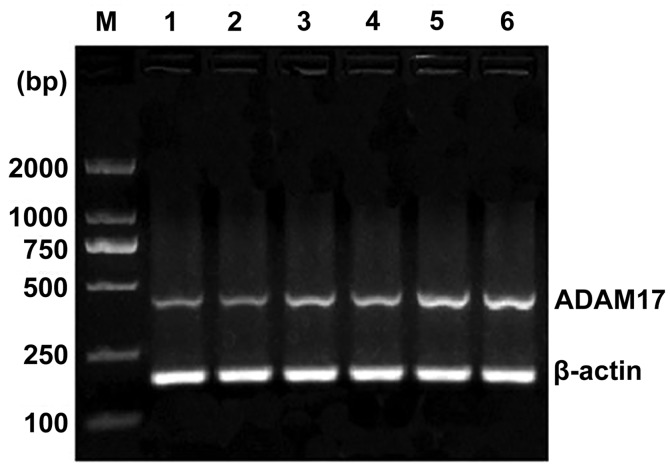
ADAM17 mRNA reverse transcription polymerase chain reaction amplification results electrophoresis. Lanes: M, DNA Marker; 1 and 2, histological grade I; 3 and 4, histological grade II; 5 and 6, histological grade III.

The length of the reference β-actin gene fragment was 218 bp. The length of ADAM17 gene fragment was 440 bp. The normal esophageal mucosa and esophageal squamous cell carcinoma had varying intensities of expression (Fig. 1). In the 50 cases of esophageal squamous cell carcinoma, the ratio of ADAM17 mRNA expression to that of β-actin was increased to 0.937±0.241 (Table I), compared with the ratio of expressions in normal esophageal mucosa, which was 0.225 ± 0.077 (P<0.01). As shown in Table II, in the 50 cases of esophageal squamous cell carcinoma, ADAM17 mRNA expression was significantly higher in the lymph node metastasis group than that of the lymph node-negative group (P<0.01) (Fig. 2). In the TNM stages, the ADAM17 mRNA expression of stage I + II patients was significantly different to that of stage III + IV patients (P<0.05). Esophageal squamous cell histology revealed that the levels of ADAM17 mRNA expression in stage I, II and III was increased, however, no significant difference was determined (P>0.05) (Fig. 3). In addition, ADAM17 mRNA expression had no correlation with other clinicopathological factors, including gender and age (P>0.05).

### ADAM17 protein levels in esophageal squamous cell carcinoma

**Figure 4:**
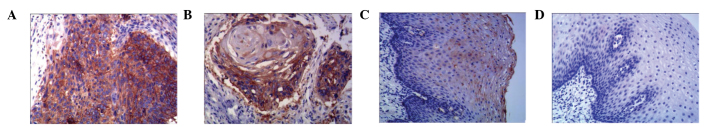
A disintegrin and metalloproteinase 17 protein in esophageal squamous cell carcinoma and adjacent tissues stained using streptavidin peroxidase conjugated immunohistochemistry (magnification, ×100). (A) Squamous cell carcinoma (+++); (B) squamous cell carcinoma (++); (C) adjacent tissues (+); (D) adjacent tissues (−).

Following SP immunohistochemical staining, ADAM17 expression in esophageal squamous cell carcinoma was defined as brown or brown-black particles located in the cell cytoplasm (Fig. 4). Of the 80 cases of esophageal squamous cell carcinoma (Table I), 53 cases demonstrated positive ADAM17 protein expression (66.25%) and five cases had positive expression in the corresponding normal tissues (6.25%), a statistically significant difference (P<0.01). The percentage of positive ADAM17 protein expression in the lymph node metastasis group was significantly higher than that of the lymph node-negative group (P<0.05). In TNM stages, the rate of positive ADAM17 protein expression in stage I + II patients was significantly increased compared to that of III + IV patients (P<0.05). The rate of positive ADAM17 protein expression increased with the stages of esophageal histology, however, this change was not found to be statistically significant (P>0.05). The ADAM17 protein expression showed no correlation with other clinicopathological factors, including gender and age (P>0.05, Table II). Of the 80 cases of esophageal squamous cell carcinoma, 53 cases showed positive ADAM17 expression, 40 of which had positive EGFR expression, while of the 27 cases that were negative for ADAM17 expression, 9 cases had positive EGFR expression. Statistical analysis showed that the EGFR positive expression rate in the ADAM17 positive expression group was significantly higher than the of the ADAM17 negative expression group (P<0.01). Column correlation coefficient analysis showed that ADAM17 expression and EGFR expression were positively correlated (P<0.01, Table III).

### Prognostic significance of ADAM17 and EGFR expression in esophageal squamous cell carcinoma

**Figure 5:**
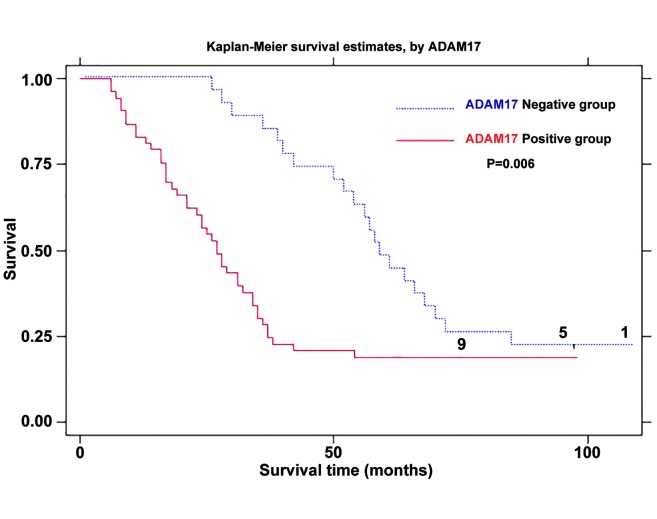
ADAM17 the Kaplan-Meier survival curves. ADAM17, a disintegrin and metalloproteinase 17.

The follow-up data showed that the 1, 3 and 5-year survival rates in esophageal squamous cell carcinoma were 85.94, 34.38 and 11%, respectively. Through the follow-up data of esophageal squamous cell carcinoma patients, a univariate logrank survey showed that ADAM17 and EGFR were prognostic factors affecting esophageal squamous cell carcinoma (All P<0.05, Table IV). Further ADAM17 Kaplan-Meier survival curves showed that the survival rate in the ADAM17 positive group was significantly lower than those in ADAM17 negative group (P<0.01) (Fig. 5). The multivariate Cox proportional hazards model analysis showed that ADAM17, EGFR, lymph node metastasis and TNM stage all had independent prognostic significance (all P<0.05, Table V). This indicates that ADAM17 and EGFR expression may increase the risk of esophageal cancer mortality.

## Discussion

China has a high incidence of esophageal cancer. The majority of patients do not exhibit early symptoms, and a number of them do not receive treatment in the late phase. In the early and mid terms of esophageal cancer, the most common treatments are surgery or radiotherapy and supplemented with chemotherapy treatment. For late phase patients, chemotherapy was used as a palliative treatment. With the progress in tumor molecular biology, the tumors molecular targeted therapy acting on tumor cells had target selectivity, efficiency, low toxicity and other advantages. It achieved notable results in breast cancer and non-small cell lung cancer therapy. Currently, looking for the effective molecular targets in esophageal cancer treatment became a research hotspot of esophageal cancer.

The ADAM family includes a set of transmembrane secretory proteins composed of multiple domains. Their most clear effect is the release of certain important biological ligands, including tumor necrosis factor α, epidermal growth factor and transforming growth factor α. Since these ligands involved in tumor formation and progression, it can be inferred that the specific molecular ADAMs associated with the release of these ligands are involved in the malignant process (1,2). ADAMl7 is an ADAM superfamily also known as TACE. Le *et al* (3) found that in ADAM17 deficient mice, T cells lost almost all of their ability to release tumor necrosis factor-α. and that ADAM17 was likely to be physiological shedding enzyme of tumor necrosis factor-α; the function of the ADAMl7 protein was that it hydrolyzes and releases precursor cell surface proteins. It transversely activates the cell surface molecules of the cell signaling pathway to change the signal transmission. The biological behaviors of tumor cells, including tumor angiogenesis, extracellular matrix degradation, remodeling and cell adhesion functions, reflect the biological behavior of tumor cells and cellular signal transduction, and exhibited a close association with ADAMl7 function (4,5).

In recent years, studies found the high expression of ADAM17 in a variety of human tumors, it reflected the degree of malignancy, promoted tumor invasion and metastasis process, it was associated with prognosis of cancer in patients. McGowan *et al* (6) reported the association between ADAM17 and breast cancer in MRNA and protein levels respectively. They found that ADAM17 mRNA expression in breast cancer tissue was positively correlated with the number of lymph node metastasis. It proved that the ADAM17 mRNA was involved in breast carcinogenesis and progression, the high expression of ADAM17 will increase the invasiveness and spreading ability of MCF-7 breast cancer cells *in vitro*. ADAM17 can shed amphiregulin, transforming growth factor α and other EGFR ligands, it can also activate the EGFR to thereby improve the lung cancer cell proliferation and cell motility capacity (7). ADAM17 expression increased in gastric cancer cells. ADAM17 promoted cancer cell proliferation through shedding EGFR ligands (8). Ding *et al* (9) found that the HCC ADAM17 mRNA expression was significantly higher than that in the surrounding liver tissue. It was closely associated with degree of differentiation in liver cancer suggesting that the ADAM17 was associated with the development of liver cancer. Kornfeld *et al* (10) reported that ADAM17 can activate the EGFR through the release of regulatory proteins in two ways to thereby enhance the proliferation and invasion of head and neck squamous cell carcinoma. Hinsley *et al* (11) confirmed that ADAM17-mediated release of EGFR ligands triggered the head and neck cancer cell migration and were involved in formation of metastatic squamous cell carcinoma. ADAM17 and esophageal cancer studies were rarely reported, Sakamoto, etc. (12) used the RT-PCR and western blot analysis protein electrophoresis methods, they detected the elevated ADAMTS16 protein expression in esophageal tissues. In the medium TE5 esophageal cancer cell lines, ADAMTS16 protein levels were detected. By blocking the ADAMTS16, the growth and invasion of TE5 esophageal cancer cell can be inhibited.

The present study used the RT-PCR and SP methods, the results showing that ADAMl7 mRNA and protein exhibited varying intensity expression of normal esophageal mucosa and esophageal squamous cell carcinoma. However, ADAM17 mRNA and protein expression in esophageal squamous cell carcinoma was significantly higher than that in the normal esophageal mucosa. ADAMl7 mRNA and protein overexpression suggested gene mutation occurs in the process of esophageal carcinoma. ADAMl7 mRNA exhibited a certain association with the occurrence and development of esophageal squamous cell carcinoma. This provided a reference for the diagnosis and differential diagnosis of benign and malignant lesions in esophageal cancer.

The study also showed that ADAM17 mRNA expression and protein expression positive rate increased with the I, II, III stage of esophageal squamous cell histology. However, there was no statistically significant difference. ADAMl7 mRNA expression and protein positive expression rate in lymph node metastasis group of esophageal squamous cell carcinoma were significantly higher than that in the lymph node-negative group. With regard to TNM staging, ADAM17 mRNA expression and protein expression rate in patients at stages I and II were significantly different from those in patients at stages III and IV. This suggested that ADAMl7 mRNA may be associated with uncontrolled proliferation of esophageal squamous cell cancer and it may be involved in the invasion, metastasis and distant dissemination mechanisms of esophageal squamous cell carcinoma. The follow-up data showed that the esophageal squamous cell carcinoma ADAM17 expression was an important prognostic factor affecting the esophageal squamous cell carcinoma, it had independent prognostic significance as EGFR, lymph node metastasis and TNM stage. This indicated that high ADAMl7 mRNA and protein expression may become an marker for metastasis and prognosis in esophageal squamous cell carcinoma.

Lorenzen *et al* (5) found that ADAMl7 can mediate the release of specific EGFR ligands, the latter binding with EGF which activates EGFR, resulting in the epithelial hyperplasia disorder developing into cancer. Previous studies found that selective inhibitors of ADAM17 protease can block the tumor cell EGFR pathway (5,8,13). Currently the EGFR signaling pathway is a known target of targeted anticancer drugs (14). Certain drugs which use EGFR as the therapeutic target, including Erbitux^®^, are already on the market (15). The present study showed that ADAM17 expression had a positive correlation with EGFR expression indicating that in patients with esophageal squamous cell carcinoma, certain target EGFR drugs produce tolerance. ADAMl7-mediated release of specific EGFR ligands can be altered for clinical treatment. It can be envisaged that as a target for targeted therapy, ADAM17 may be a good supplement for the existing treatment of EGFR targeted therapy.

## Figures and Tables

**Table I:** ADAM17 mRNA and protein levels in esophageal squamous cell carcinoma.

	RT-PCR			SP			
		
Group	Cases (n)	ADAM17	P-value	Cases (n)	Negative (%)	Positive (%)	P-value
Esophageal squamous cell carcinoma	50	0.937±0.241	<0.001	80	27 (33.75)	53 (66.25)	<0.001
Normal esophageal	50	0.225±0.077		80	75 (93.75)	5 (6.25)	

ADAM17, a disintegrin and metalloproteinase 17; RT-PCR, reverse transcription polymerase chain reaction; SP, streptavidin peroxidase conjugated immunohistochemistry. ADAM17 levels are represented as a ration of its expression to that of β-actin. Data are presented as the mean ± standard deviation.

**Table II:** ADAM17 mRNA and protein in esophageal squamous cell carcinoma.

	RT-PCR			SP			
		
Category	Cases (n)	ADAM17	P-value	Cases (n)	Negative (%)	Positive (%)	P-value
Gender			0.766				0.518
Male	33	0.947±0.276		57	18 (31.58)	39 (68.42)	
Female	17	0.923±0.252		23	9 (39.13)	14 (60.87)	
Age (years)			0.369				0.297
≤60	23	0.902±0.314		35	14 (40.00)	21 (60.00)	
>60	27	0.974±0.247		45	13 (28.89)	32 (71.11)	
Histological grade			0.148				0.150
I	10	0.852±0.184		15	8 (53.33)	7 (46.67)	
II	26	0.923±0.218		43	14 (32.56)	29 (67.44)	
III	14	1.241±0.141		22	5 (22.73)	17 (77.27)	
Lymph node metastasis			0.000				0.006
Absence	30	0.884±0.184		45	21 (46.67)	24 (53.33)	
Presence	20	1.172±0.249		35	6 (17.14)	29 (82.86)	
TNM stage^a^			0.011				0.009
I + II	29	0.894±0.131		43	20 (46.51)	23 (53.49)	
III + IV	21	1.024±0.215		37	7 (18.92)	30 (81.08)	

a Since TNM stages I and IV had fewer cases, they were merged into the other two groups.

RT-PCR, reverse transcription polymerase chain reaction; SP, streptavidin peroxidase conjugated immunohistochemistry; ADAM17, a disintegrin and metalloproteinase. ADAM17 levels are represented as a ration of its expression to that of β-actin. Data are presented as the mean ± standard deviation.

**Table III:** ADAM17 and EGFR expression in esophageal squamous cell carcinoma compared.

		EGFR				
					
ADAM17	Cases (n)	Negative (%)	Positive (%)	P-value	Cramer’s V	γ
Negative	27	18 (66.67)	9 (33.33)	<0.001	0.409	0.720
Positive	53	13 (24.53)	40 (75.47)			

ADAM17, a disintegrin and metalloproteinase; EGFR, epidermal growth factor receptor.

**Table IV:** ADAM17 and EGFR expression in esophageal squamous cell carcinoma and patient survival data (Logrank test).

Category	Cases (n)	Average survival time (month)	95% CI	χ^2^	P-value
ADAM17 expression					
Negative	27	64.444±5.191	54.271, 74.618	7.56	0.006
Positive	53	37.377±4.247	29.053, 45.702		
EGFR expression					
Negative	31	59.349±4.664	50.208, 68.491	5.28	0.022
Positive	49	39.755±5.109	29.740, 49.770		

CI, confidence interval; EGFR, epidermal growth factor receptor; ADAM17, a disintegrin and metalloproteinase 17.

**Table V:** Multivariate survival analysis of esophageal cancer (Cox proportional hazards model).

Clinicopathological factors	Variable coefficient	Standard error	Z-statistic	P-value	95% CI
Age	−0.122	0.252	−0.491	0.627	−0.615, 0.371
Gender	−0.311	0.285	−1.094	0.275	−0.869, 0.247
Classification	0.041	0.178	0.232	0.819	−0.307, 0.389
Lymph node metastasis	0.601	0.243	2.375	0.018	0.104, 1.097
TNM staging	0.526	0.226	2.085	0.037	0.031, 1.021
EGFR	0.585	0.260	2.254	0.024	0.076, 1.095
ADAM17	0.724	0.271	2.683	0.007	0.194, 1.254

CI, confidence interval; TNM, tumor node metastasis; EGFR, epidermal growth factor receptor; ADAM17, a disintegrin and metalloproteinase 17.
